# Immunogenicity of CRISPR therapeutics—Critical considerations for clinical translation

**DOI:** 10.3389/fbioe.2023.1138596

**Published:** 2023-02-16

**Authors:** Radwa Ewaisha, Karen S. Anderson

**Affiliations:** ^1^ Department of Microbiology and Immunology, Faculty of Pharmacy, Alexandria University, Alexandria, Egypt; ^2^ Department of Microbiology and Immunology, School of Pharmacy, Newgiza University, Newgiza, Egypt; ^3^ Center for Personalized Diagnostics, Biodesign Institute, Arizona State University, Tempe, AZ, United States

**Keywords:** CRISPR-Cas, immunogenicity, clinical translation, Cas9, genome editing, gene therapy, clinical trials

## Abstract

CRISPR offers new hope for many patients and promises to transform the way we think of future therapies. Ensuring safety of CRISPR therapeutics is a top priority for clinical translation and specific recommendations have been recently released by the FDA. Rapid progress in the preclinical and clinical development of CRISPR therapeutics leverages years of experience with gene therapy successes and failures. Adverse events due to immunogenicity have been a major setback that has impacted the field of gene therapy. As several *in vivo* CRISPR clinical trials make progress, the challenge of immunogenicity remains a significant roadblock to the clinical availability and utility of CRISPR therapeutics. In this review, we examine what is currently known about the immunogenicity of CRISPR therapeutics and discuss several considerations to mitigate immunogenicity for the design of safe and clinically translatable CRISPR therapeutics.

## 1 Introduction

Since the discovery of the Clustered Regularly Interspaced Short Palindromic Repeat (CRISPR)-Cas9 technology, there has been rapid progress in the development of personalized gene therapeutics with a promise to treat complex diseases. CRISPR has the advantages of high efficiency, targetability, accuracy, ease of use, and limited off-target effects, over other gene editing tools. The number of ongoing CRISPR-based gene-editing clinical trials is markedly increasing. These new potential therapies are intended to treat cancer, genetic disorders, metabolic disorders, and infectious diseases, among others. While most trials involve *ex vivo* gene editing followed by cell transplantation into the patient, *in vivo* delivery of CRISPR gene-editing components is currently underway (Gillmore et al., 2021; Intellia Therapeutics, 2021; Excision BioTherapeutics, 2022).

The bacterial origin of the CRISPR system has raised concerns over the potential immunogenicity of CRISPR-based therapeutics. We and others have previously reported the detection of pre-existing immunity to the Cas9 protein in healthy adults (Simhadri et al., 2018; Charlesworth et al., 2019; Ferdosi et al., 2019; Wagner et al., 2019; Tang et al., 2022). Immunity can also be induced in naïve individuals upon repeated administration, whether against the gene delivery vector or the gene editing components. Immunogenicity, while not necessarily consequential, can impact both therapeutic safety and efficacy. A severe adverse immune response to an administered gene therapeutic led to the death of 18-year-old Jesse Gelsinger in 1999 (Marshall, 1999) and slowed down progress in gene therapy for many years. As gene therapy is revisited with CRISPR, the question of pre-existing and acquired immunogenicity stands out as a potential roadblock to the clinical availability and utility of CRISPR therapeutics. Here, we review what is currently known about the immunogenicity of CRISPR therapeutics and discuss important factors that need to be considered for successful clinical translation.

## 2 Immune response to CRISPR therapeutics

CRISPR therapeutics are composed of a CRISPR effector protein such as Cas9, guided to target nucleic acid sequences with a guide RNA (gRNA), and delivered using a viral or non-viral delivery vector. Each of these components can induce an immune response upon administration. First, CRISPR effectors are large foreign proteins derived from bacteria or archaea and can induce specific effector and memory adaptive immune responses. Second, gRNAs can trigger an innate immune response through interacting with pattern recognition receptors (Chew, 2018). *In vitro* transcribed 5′-triphosphate gRNAs but not chemically synthesized 5′-hydroxylated gRNAs have been shown to trigger an innate immune response leading to cytotoxicity (Kim et al., 2018). Thus, while gRNA modification may be an effective strategy, gRNA immunogenicity needs to be taken into consideration, since it might not always be feasible to completely eliminate gRNA immune recognition while retaining the required sequence and structure (Chew, 2018). Third, adeno-associated viruses (AAV) that are widely used for delivery of gene therapeutics, although less immunogenic than other viral vectors, are targets of both pre-existing and inducible adaptive immune responses that may be cross-reactive between different serotypes (Verdera et al., 2020). In this review, we focus on the immunogenicity of CRISPR effector proteins and lessons learned from pre-clinical and clinical studies.

### 2.1 Pre-existing immune responses to CRISPR effector proteins

The two most widely studied Cas9 orthologs, SpCas9 and SaCas9, are derived from the two ubiquitous bacteria *Streptococcus pyogenes*, a common bacterial pathogen that causes more than 700 million infections annually worldwide (Ralph and Carapetis, 2013), and *Staphylococcus aureus*, a common human commensal and the leading cause of skin and soft tissue infections (Tong et al., 2015), respectively. Pre-existing adaptive immune responses to different CRISPR effector proteins have been detected in the general population (Simhadri et al., 2018; Charlesworth et al., 2019; Ferdosi et al., 2019; Wagner et al., 2019; Tang et al., 2022; Toral et al., 2022). Immune responses can also be induced in naïve individuals upon administration of therapy. Both B cell and T cell specific immune responses have been reported in mice following the administration of SpCas9 (Wang et al., 2015; Chew et al., 2016) and SaCas9 (Nelson et al., 2019).

Our group reported a prevalence of pre-existing anti-SpCas9 antibodies of at least 5% (Ferdosi et al., 2019) in the general population, consistent with (2.5%) Simhadri et al. (2018), but different from (58%) Charlesworth et al. (2019) and (95%) Tang et al. (2022) (Table 1). Pre-existing antibodies against SaCas9 were prevalent in 10% (Simhadri et al., 2018), 4.8% (Shen et al., 2022), and 95% (Tang et al., 2022) of healthy donors in three studies. Surprisingly, pre-existing antibodies against RfxCas13d from *Ruminococcus flavefaciens*, not known to colonize humans, have been reported at a prevalence similar to anti-SpCas9 and anti-SaCas9 reported by the same study (Tang et al., 2022). Interestingly, one study detected pre-existing anti-SpCas9 antibodies in all adult dogs and detected modest levels of maternal antibodies in newborn puppies, which dropped within 6 weeks of birth (Hakim et al., 2021).

**TABLE 1:** Studies reporting pre-existing immunity to CRISPR effector proteins in healthy individuals.

Study	CRISPR effector	Source organism	Individuals with pre-existing adaptive immune responses (%)		Number of individuals tested	Proposed strategy for mitigation of immunogenicity
			Abs	T cell		
Simhadri et al. (2018)	Cas9	*S. pyogenes*	2.5%	N/A	200	N/A
	Cas9	*S. aureus*	10%	N/A		
Charlesworth et al. (2019)	Cas9	*S. pyogenes*	58%	67%	125, 18 (Abs, T cell)	N/A
	Cas9	*S. aureus*	78%	78%		
Wagner et al. (2019)	Cas9	*S. pyogenes*	N/A	95%	45	Adoptive transfer or stimulation of T_reg_ cells
	Cas9	*S. aureus*	N/A	100%	6	
	Cas12a	*Acidaminococcus* sp.	N/A	100%	6	
Ferdosi et al. (2019)	Cas9	*S. pyogenes*	5%	83%	143, 12 (Abs, T cell)	Engineered SpCas9 with immunosilenced epitopes
Stadtmauer et al. (2020)*	Cas9	*S. pyogenes*	0%	66.7%	3	*Ex vivo* gene editing and confirmation of minimal levels of Cas9 protein prior to infusion
Tang et al. (2022)	Cas13d	*Ruminococcus flavefaciens*	89%	96%/100% (CD8^+^/CD4^+^)	19, 24 (Abs, T cell)	N/A
	Cas9	*S. pyogenes*	95%	96%/92% (CD8^+^/CD4^+^)		
	Cas9	*S. aureus*	95%	96%/88% (CD8^+^/CD4^+^)		
Shen et al. (2022)	Cas9	*S. aureus*	4.8%	70%	123, 10 (Abs, T cell)	Engineered SaCas9 with immunosilenced epitopes
Toral et al. (2022)	Cas9	*S. pyogenes*	100%/15% (paired serum/vitreous fluid)	N/A	13	N/A
	Cas9	*S. aureus*	100%/15% (paired serum/vitreous fluid)	N/A		

*Clinical trial. Abs, antibodies.

Several studies have detected pre-existing cellular immunity against SpCas9 and SaCas9 in most healthy individuals tested, with the percentage of responsive donors ranging from 57%–95% (Summarized in Table 1) (Charlesworth et al., 2019; Ferdosi et al., 2019; Wagner et al., 2019; Shen et al., 2022). Similarly, pre-existing T cell responses to Cas12a from *Acidaminococcus sp.* (Wagner et al., 2019) and RfxCas13d (Tang et al., 2022), have been detected in healthy individuals at comparable frequencies to SpCas9 and SaCas9 responses. Sequence similarity between Cas9 orthologs from different bacteria and sequence similarity with other non-CRISPR-related bacterial proteins could contribute to widespread pre-existing adaptive immune responses even to CRISPR systems from less ubiquitous prokaryotes. *Acidaminococcus sp.* Cas12a shares 38% sequence homology to SpCas9 (Wagner et al., 2019). Similarly, pre-existing immune responses to RfxCas13d could be a result of the fact that *Ruminococcus flavefaciens*, a bovine commensal, shares close sequence homology to Cas13d proteins from other *Ruminococcus* species that colonize the human gut (Tang et al., 2022).

Besides known protein sequence homology, Cas9-specific immunodominant epitopes were found to be shared between different bacterial species. Identified SpCas9 MHC class I-restricted human leukocyte antigen (HLA) A*02:01 epitopes were similar to sequences on Cas9 orthologs from other bacterial species and at least one was similar to a peptide from a *Neisseria meningitidis* non-CRISPR-related protein (Ferdosi et al., 2019). However, pre-existing immune responses were also detected against RfxCas13d peptides that are not recognized to be homologous to peptides from known bacterial proteins (Tang et al., 2022). While the diversity of CRISPR systems provides an opportunity to explore a diverse and versatile CRISPR toolbox, it also adds the need to study the immunogenicity of each CRISPR system individually and assess safety before clinical translation.

### 2.2 Significance of the immune response to CRISPR therapeutics

Specific immune responses to CRISPR-based therapeutics do not necessarily imply an adverse or unfavorable response in treated individuals. However, evidence from preclinical studies suggests that specific immunity can potentially lead to failure of the intended gene editing therapy or even to a damaging immune reaction (Wang et al., 2015; Li et al., 2020). One study that delivered SpCas9 in an adenovirus vector to the liver in mice reported success of Pten gene editing despite significant inflammatory responses (Wang et al., 2015). In another study, a CD8^+^ T cell response was elicited in mice that had been immunized against SaCas9 prior to the transduction of hepatocytes with CRISPR-Cas9 packaged in an adeno-associated virus (AAV) vector, resulting in hepatocyte apoptosis and failure of the genome editing procedure (Gough and Gersbach, 2020; Li et al., 2020). Chew and co-authors reported that Cas9 expression in mouse muscles resulted in Cas9-driven lymphocyte infiltration in the muscle tissue and draining lymph nodes, which was not seen when the AAV vector without the Cas9 sequence was administered (Chew et al., 2016). These histopathological changes were observed regardless of the delivery method, although they were more prominent with muscle electroporation than with AAV delivery (Chew et al., 2016).

Ajina et al. (2019) showed that tumors expressing SpCas9 were rejected by a Cas9-specific T cell response in immunocompetent but not immunodeficient mice. B cells expressing SpCas9 were lysed in a dose-dependent manner when challenged *in vitro* with autologous SpCas9-specific T_eff_ cells, suggesting that higher levels of Cas9 expression for gene editing are more likely to trigger a deleterious immune response (Wagner et al., 2019). Taken together, these findings have significant implications for therapeutic strategies that require persistent *in vivo* Cas9 gene expression or repeated gene therapy dosing to achieve clinically useful results.

## 3 Assessment of the immunogenicity of CRISPR therapeutics—*In silico* prediction

With the diversity of CRISPR effector orthologs and HLA haplotypes, *in silico* prediction algorithms represent a more time and cost-efficient method for systematic assessment of immunogenicity and identification of epitopes for immunosilencing. The initial computational identification is then followed by *in vitro* and/or *in vivo* assays for confirmation. For T cell epitopes, multiple factors determine whether a given peptide will be immunogenic or immunodominant. These include proteasomal cleavage patterns; peptide binding affinity to MHC molecules and stability of the complex formed; the availability of a T cell repertoire that recognizes the peptide-MHC complex; and other factors that are not fully understood (Ducret et al., 2022). Most *in silico* prediction algorithms depend on the assessment of binding affinity of MHC molecules to linear 9- to 15-mer epitopes on the protein under study and the protein’s cleavage pattern (Moutaftsi et al., 2006; Nielsen et al., 2007).

There is a need for improved immunogenicity prediction algorithms that address current limitations to develop clinically translatable CRISPR therapeutics for universal use. Epitopes predicted using algorithms that depend solely on HLA affinity include a considerable number of good binders that are not found to be immunogenic upon experimental validation (Newell et al., 2013). Understanding peptide properties that contribute to immune recognition can lead to improved epitope prediction (Chowell et al., 2015). MHC polymorphism adds another level of complexity to the process of identification and validation of immunogenic epitopes. The most common HLA alleles have been more widely studied with more data available for algorithm training (Peters et al., 2020; Zinsli et al., 2021). MHC class I prediction tools have seen more rapid progress than MHC class II algorithms. This is because MHC class II molecules have an open-ended binding groove that accommodates peptides of varying length, which complicates prediction (Peters et al., 2020; Zinsli et al., 2021). Public availability of epitope datasets such as the Immune Epitope Database (IEDB) (Vita et al., 2015) has accelerated progress in machine learning prediction tools. These advancements in prediction tools are expected to help with the development of less immunogenic CRISPR therapeutics.

## 4 Strategies for circumventing CRISPR effector-specific immune responses

Over the last few decades, several strategies have been developed for minimizing the immune response to gene therapy and foreign protein therapeutics, some of which have been adopted for CRISPR either individually or in combination (Table 2; reviewed in Chew, 2018; Mehta and Merkel, 2020). These include adoptive transfer of T_reg_ cells (Wagner et al., 2019), protein immunosilencing by masking of immunogenic epitopes (Ferdosi et al., 2019), and the use of immune orthogonal CRISPR effector orthologs for repeat dosing (Moreno et al., 2019). Several clinical trials have delivered CRISPR components *ex vivo* which is expected to trigger minimal immune responses upon adoptive transfer to the patient, especially with transient CRISPR effector expression (Stadtmauer et al., 2020). Other strategies that have been employed by clinical trials include CRISPR delivery to immune privileged sites (Editas Medicine, 2022), the administration of immunosuppressive drugs prior to systemic CRISPR delivery (Gillmore et al., 2021), and CRISPR administration in immunocompromised individuals (Xu et al., 2019; Lu et al., 2020; Stadtmauer et al., 2020).

**TABLE 2:** Strategies for mitigating CRISPR effector-specific immune responses.

Strategy	Tested in	Results reported	Reference(s)
Adoptive transfer or stimulation of specific T_reg_ cells	Healthy donor PBMCs	SpCas9-specific T_reg_ cells inhibited SpCas9-specific T_eff_ cells	Wagner et al. (2019)
Immune orthogonal CRISPR effector protein orthologs	Mice	Higher gene editing efficiency in repetitive doses than when the same CRISPR effectors were used	Moreno et al. (2019)
Engineered immunosilenced epitopes of CRISPR effector protein	Healthy donor PBMCs	Abolished (Ferdosi et al., 2019) or reduced (Shen et al., 2022) immune recognition with a single amino acid substitution while retaining function and specificity	SpCas9 (Ferdosi et al., 2019)
			SaCas9 (Shen et al., 2022)
Immune privileged sites	Human eye (clinical trial for LCA10)	No detectable Cas9-specific B cell or T cell responses and mostly mild adverse events	Editas Medicine (2022)
*Ex vivo* gene editing	Clinical trial for refractory cancer	All 3 patients had no pre-existing Cas9-specific antibodies; no specific antibodies developed after treatment; minimal remnant Cas9 detected in cell products before infusion	Stadtmauer et al. (2020)
Self-limiting CRISPR effector protein expression	293FT cell line	Transient SpCas9 expression that still allowed the intended nuclease activity	Ruan et al. (2017)
Immune suppression	Clinical trial for transthyretin amyloidosis	Glucocorticoid and antihistamine type 1 and type 2 administered; no serious adverse events reported; successful and long-lasting TTR protein reduction	Gillmore et al. (2021)
Use in immunocompromised individuals	Clinical trials for cancer	No significant immunological adverse events in an *ex vivo* approach	Xu et al. (2019), Lu et al. (2020), Stadtmauer et al. (2020)

PBMCs, peripheral blood mononuclear cells; LCA10, Leber congenital amaurosis type 10.

## 5 Considerations to mitigate immunogenicity for clinical translation of CRISPR therapeutics

**FIGURE 1:**
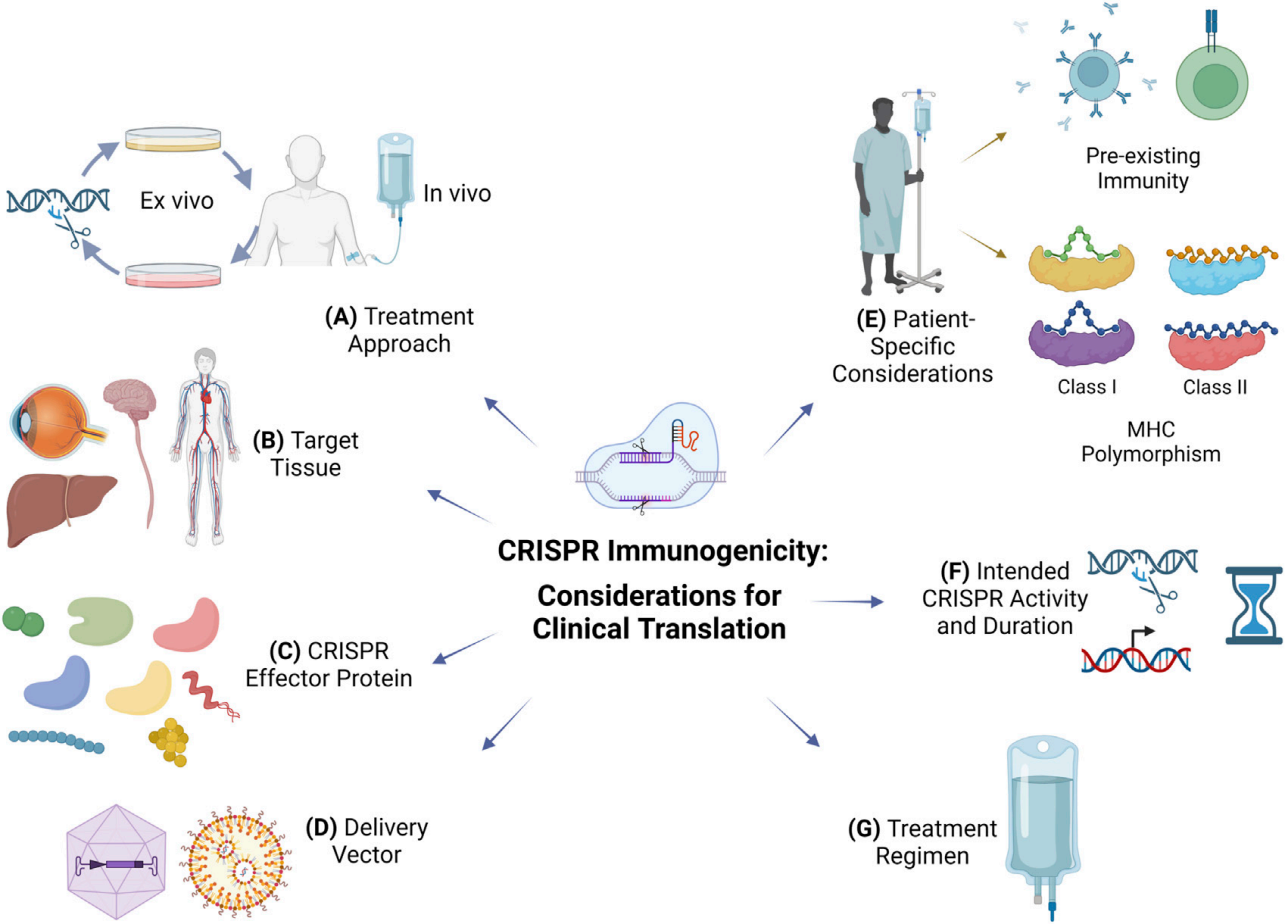
Considerations to mitigate immunogenicity for clinical translation of CRISPR therapeutics. **(A)** Treatment approach: *ex vivo* gene editing poses a smaller risk of immunogenicity than *in vivo* administration but has limited utility for diseases requiring systemic delivery of CRISPR components. **(B)** Target tissue: CRISPR administration in immune-privileged sites may limit immunogenicity. **(C)** CRISPR effector protein: non-cross-reactive CRISPR effector protein orthologs from non-ubiquitous bacteria or archaea can be considered for repeated dosing or in individuals with pre-existing immunity. **(D)** Delivery vector: viral vectors are more efficient than non-viral vectors such as lipid nanoparticles but immunogenicity and ortholog cross-reactivity could limit repeated administration; viral vector tissue tropism can be used for targeting, which reduces the dose and systemic immune responses. **(E)** Patient-specific considerations: include immune status, pre-existing B and T cell immune responses, and MHC polymorphism which dictates immunodominant MHC class I and class II epitopes of various CRISPR effectors. **(F)** Intended CRISPR activity and duration: control of CRISPR duration of action could limit specific immune responses; the intended use of CRISPR for its nuclease or transcriptional modulation activity will dictate its persistence, duration, and immunogenicity; immunogenicity against the transgene product is possible in CRISPR therapeutics intended for targeted insertion of a therapeutic transgene. **(G)** Treatment regimen: co-administration of immunosuppressive drugs or adoptive transfer of CRISPR effector-specific T_reg_ cells can limit specific immune responses.

CRISPR offers new hope for many patients and promises to transform the way we think of future therapies. Ensuring safety is a top priority for clinical translation. The FDA has recently released a draft guidance for Investigational New Drugs (INDs) that involve genome editing (Food and Drug Administration, 2022). The guidance recommends the evaluation of off-target effects using multiple orthogonal methods as well as immunogenicity of the gene editing components and the expressed gene product. Rapid progress in the preclinical and clinical development of CRISPR therapeutics leverages years of experience with gene therapy successes and failures. Immunogenicity was a major setback that brought a halt to gene therapy for a while (Marshall, 1999). Low immunogenicity is not a property that can be easily added to the finished therapeutic product, and it needs to be considered early in the development process. Here, we identify and discuss several considerations for the design of clinically translatable CRISPR therapeutics to mitigate immunogenicity (Figure 1).

### 5.1 Treatment approach

Most CRISPR clinical trials to date use *ex vivo* gene editing approaches, where the immunogenicity of CRISPR therapeutics may not be a substantial concern. The approach depends on the temporary *in vitro* expression of the CRISPR effector protein in autologous cells, which entails rapid elimination of the expressed CRISPR effector protein before infusion to the patient (Stadtmauer et al., 2020). The number of *ex vivo* CRISPR clinical trials is rapidly expanding (Uddin et al., 2020) and they are aimed at a spectrum of diseases, from sickle cell anemia and thalassemia (Frangoul et al., 2021) to HIV (Xu et al., 2019) and cancer (Lu et al., 2020; Stadtmauer et al., 2020; Uddin et al., 2020). Persistence of gene-edited cells has been reported (Xu et al., 2019; Lu et al., 2020; Stadtmauer et al., 2020), even up to 53 weeks post-treatment in one patient (Lu et al., 2020). Stadtmauer et al. (2020) confirmed minimal remnant Cas9 in edited cells prior to infusion and reported no immunogenicity-related events despite having detected pre-existing T cell responses in two of three patients. Lack of adverse immunological reactions in some of these clinical trials could be because recruited individuals were immunocompromised with a history of cancer treatment.

*Ex vivo* gene-editing has limited utility for many genetic diseases that require systemic delivery of gene-editing components. With safety concerns including CRISPR immunogenicity, off-target effects, and potential genome integration, the number of *in vivo* gene-editing trials has been advancing at a slower pace. To help minimize immunogenicity with *in vivo* treatments, it is important to consider the nature of the disease, the required gene editing procedure, and delivery vehicle specificity and persistence. Numerous applications only require transient and localized expression of CRISPR effectors, achieved through local administration in an appropriate delivery vehicle, which may reduce the risk of adverse systemic immune reactions. One study that delivered Cas9 locally or systemically to the muscles to restore dystrophin expression in canine models of Duchenne muscular dystrophy reported Cas9-specific humoral and cellular immune responses with both modes of delivery (Hakim et al., 2021). Even with high dose prednisolone administration, the authors report abundant T cell infiltration, elevation of cytokines in the muscles, and loss of dystrophin expression (Hakim et al., 2021).

Local delivery of CRISPR components has been attempted in clinical trials, although detailed findings have yet to be published. A phase 1 clinical trial that delivered CRISPR-Cas3 recombinant bacteriophage (LBP-EC01) *via* the intraurethral route for urinary tract infections (UTIs) has been completed (Locus Biosciences, 2022). No drug-related adverse events were reported (Locus Biosciences, 2022) and a phase 2/3 trial is currently underway (Locus Biosciences, 2021). LBP-EC01 is a bacteriophage with CRISPR-Cas3 that targets *E. coli*-specific sequences to enhance the phage bactericidal effect. Even though phage therapy is not currently an approved treatment, multiple non-CRISPR-related clinical trials have evaluated its safety and efficacy in UTIs *via* local delivery and reported a favorable safety profile (Leitner et al., 2017; Ujmajuridze et al., 2018; Leitner et al., 2021).

The first clinical trial involving systemic delivery of CRISPR components used SpCas9 and a sgRNA targeting TTR (NTLA-2000) to reduce the production of misfolded transthyretin protein in hereditary transthyretin amyloidosis (Gillmore et al., 2021). In six patients who received NTLA-2000, adverse reactions were mild, and one patient had an infusion-related reaction. Prior to infusion, patients received glucocorticoid and histamine type 1 and type 2 blockers to minimize inflammatory reactions. Delivery was achieved by proprietary lipid nanoparticles (LNPs) with liver tropism, which minimizes both persistence of CRISPR components compared to viral vector delivery and systemic side effects. A strong, persistent, dose-dependent reduction in TTR serum concentrations was observed with a single dose, supporting the use of this approach for the treatment of hereditary transthyretin amyloidosis. The same sponsoring company is using a similar approach in a clinical trial of hereditary angioedema through systemic administration of CRISPR delivered by LNPs (NTLA-2002) that preferentially accumulate in the liver (Intellia Therapeutics, 2021). Similar to the transthyretin amyloidosis trial, interim data show only mild adverse reactions (Intellia Therapeutics, 2022). Detailed protocols have not been published to date, but it is possible that patients received an immunosuppressive regimen similar to that used in the transthyretin amyloidosis study. VERVE-101 is another *in vivo* CRISPR therapeutic delivered to hepatocytes with LNPs that is currently being tested for the treatment of familial hypercholesterolemia (Musunuru et al., 2021; Verve Therapeutics, 2023).

Current *in vivo* trials are not restricted to the use of LNPs that target the liver. A clinical trial that delivers CRISPR components systemically is ongoing for the treatment of HIV (Excision BioTherapeutics, 2022). In this trial, an adeno-associated viral vector is used to deliver sgRNAs targeting HIV sequences with the purpose of eliminating proviral DNA from cell reservoirs. Unlike hereditary transthyretin amyloidosis in which knockout of mutated genes from only a proportion of hepatocytes can be sufficient for symptomatic treatment, HIV cure requires the complete elimination of HIV DNA from all cell reservoirs. It remains to be seen if either viral vector delivery or potential repeated dosing will increase the chances of an unfavorable immune response.

### 5.2 Target tissue

Many early gene therapy attempts were designed to target tissues that have limited natural immune response (Bainbridge et al., 2008; Maguire et al., 2009; Simonelli et al., 2010; Bouquet et al., 2019). One example of these immune-privileged sites is the eye, where inflammatory responses are limited to avoid impact on vision (Benati et al., 2020; Ren et al., 2022). Another advantage is the ease of access due to peripheral location. The first *in vivo* CRISPR clinical trial targeted photoreceptors *via* subretinal injection as a therapeutic intervention for Leber congenital amaurosis type 10 (LCA10) (Editas Medicine, 2022). The approach uses a photoreceptor-tropic AAV vector to correct a mutation that gives rise to a truncated, non-functional CEP290 protein, with Cas9 expression being restricted to photoreceptor cells (Maeder et al., 2019). Initial clinical data reported no detectable Cas9-specific B cell or T cell responses and mostly mild adverse events (Editas Medicine, 2021). However, data from early gene therapy trials using intraocular delivery show that adaptive immune responses and related adverse events could be experienced in some patients (Oliveira et al., 2017; Arbabi et al., 2019). Toral et al. reported pre-existing serum antibodies against SpCas9, SaCas9, and positive control α-tetanus in all 13 individuals tested. While vitreous fluid from all 13 individuals had pre-existing antibodies against α-tetanus, only two of them had antibodies against SpCas9 and two against SaCas9, suggesting that pre-existing immunity to CRISPR effectors may be less significant in human eyes (Toral et al., 2022).

The CNS is another immune-privileged site. However, specific targeting comes with a unique set of challenges. *Ex vivo* approaches are not a viable option, and the blood brain barrier may prevent the delivery of systemically administered therapeutics including most viral vectors (Lykken et al., 2018; Duarte and Deglon, 2020). Local intraparenchymal injection has been used for the delivery of foreign gene therapy components in animal models including AAV vectors, zinc-finger nucleases and other non-self proteins (Hadaczek et al., 2009; Garriga-Canut et al., 2012; Ciesielska et al., 2013; Samaranch et al., 2014; Agustín-Pavón et al., 2016). Surprisingly, strong inflammatory responses have been reported despite the immune privilege of the CNS (Ciesielska et al., 2013; Samaranch et al., 2014; Agustín-Pavón et al., 2016). Deimmunization by host matching of zinc-finger constructs prolonged the expression of zinc-finger nucleases and repression of the targeted mutant gene in the brain (Agustín-Pavón et al., 2016). In addition to the challenges of immunogenicity, cerebral delivery in humans is an invasive procedure and efficiency is not translated in higher-order animals (Lykken et al., 2018). Tailored tropism of the viral vector is another strategy that could circumvent the blood brain barrier (Cearley et al., 2008; Foust et al., 2009), although an immune response to these vectors or to the gene editing components that they carry is not precluded.

### 5.3 CRISPR effector protein

CRISPR-Cas systems are present in about 50% of bacteria and 90% of archaea (Hille et al., 2018; Arroyo-Olarte et al., 2021). As an adaptive immune system that provides a survival advantage, an expansive number of diverse CRISPR systems and associated proteins have evolved (Makarova et al., 2020; McKay and Burgio, 2021). Early studies have focused on *S. pyogenes* and *S. aureus* CRISPR-Cas9 systems, but now other CRISPR systems are being explored for their different efficiencies, specificities, and targeting requirements (Adli, 2018; Batool et al., 2021; McKay and Burgio, 2021). This diversity may provide an opportunity to avoid pre-existing immunity by using CRISPR effectors from less ubiquitous prokaryotes such as extremophiles. Repeat dosing can also be made possible by using a different CRISPR effector protein for each dose. However, as discussed earlier, cross-reactive immune responses could exist due to sequence similarity with other CRISPR effector proteins from common bacteria (Tang et al., 2022) or with other non-CRISPR associated proteins (Ferdosi et al., 2019). One study reported that specific antibody responses to Cas9 orthologs from *S. pyogenes*, *S. aureus*, and *C. jejuni* were not cross-reactive (Moreno et al., 2019). Cross-reactivity between different CRISPR effectors will need to be individually assessed to identify orthologs that can be used for subsequent doses.

Comprehensive *in silico* mapping of both B-cell and T cell epitopes and identification of non-cross-reactive CRISPR effective orthologs is one strategy. One of the earliest studies to predict immunogenic epitopes of CRISPR effectors identified Cas9 and Cas12a T cell epitopes for 28 HLA class I and 27 class II alleles using the IEDB tool and reported epitopes that are likely to bind to multiple MHC alleles (Chew, 2018). Moreno and co-authors evaluated sequence similarities and predicted binding affinities of major histocompatibility complex (MHC) class-I-restricted and class-II-restricted epitopes among 368 CRISPR effector orthologs (Moreno et al., 2019). Of these, five Cas9 proteins were identified to have the least predicted immune cross-reactivity (immune orthogonal) and were thus further used for serial gene delivery in mice. The efficiency of gene editing in repetitive doses was higher in mice that received Cas9 and AAV orthologs with immune orthogonality than in mice that received the same CRISPR effectors used in their first dose. This approach provides an opportunity to harness the diversity of CRISPR effectors to circumvent the immune response, but highlights the current knowledge gap and the need for enhanced prediction algorithms for less common MHC alleles to enable translation into humans (Wilson and Anderson, 2019).

### 5.4 Delivery vector

A wide variety of delivery vectors have been tested for *in vivo* delivery of gene therapy components in the last few decades and of CRISPR components in the last few years (Wilbie et al., 2019; Chuang et al., 2021; Taha et al., 2022). Viral gene delivery vectors are generally more efficient than non-viral vectors but immunogenicity limits repeated administration (Wilbie et al., 2019). AAV vectors have the advantages of low immunogenicity and low risk of genome integration (Chuang et al., 2021), although a high frequency of random integration has been reported (Gil-Farina et al., 2016; Dalwadi et al., 2021). There is a chance of prolonged expression of CRISPR effectors from AAV vectors (Herzog, 2020; Shirley et al., 2020), which constitutes a safety concern. The diversity of AAV vectors available allows screening for variants that the patient does not have pre-existing immunity against and allows, at least in theory, switching to different variants for subsequent doses. However, unlike the reported immune orthogonality with different CRISPR effectors, Moreno et al. found strong epitope conservation when comparing sequence similarity and MHC binding among 167 AAV capsid orthologs, suggesting potential cross-reactivity in individuals who receive one AAV ortholog against other AAV vectors (Moreno et al., 2019). The design of vectors with specific cell-type tropism allows targeting of certain tissues (Zincarelli et al., 2008). An AAV vector that is highly efficient at targeting skeletal muscles was engineered by screening of AAV capsids modified by inserting a random peptide (Tabebordbar et al., 2021). The use of such AAV with high target cell specificity allows reduction of the required dose and of systemic immune responses. Appropriate vector selection and synthetic vector capsid design using machine learning algorithms could also minimize immunotoxicity (Wec et al., 2021).

Adenoviruses are some of the most studied viral delivery vectors for gene therapy. They have been tested in clinical trials as delivery vectors for vaccines against HIV, Zika, and Ebola (Baden et al., 2020; Pollard et al., 2021; Salisch et al., 2021). Their scalability and cost efficiency facilitated their rapid development and large-scale distribution as vectors for vaccines against SARS-CoV-2 (Jacob-Dolan and Barouch, 2022). They have the advantage of large packaging capacity (Ismail et al., 2018; Asmamaw Mengstie, 2022) and third generation vectors allow the delivery of large CRISPR cargo in one viral vector (Brescia et al., 2020; Tasca et al., 2020). Because exposure to adenoviruses is common, pre-existing immunity is prevalent in the general population and cross-reactivity among different serotypes is frequently observed (Lee et al., 2017). CRISPR/Cas9 delivered in an adenovirus vector in mice allowed knock-in of human alpha-1-antitrypsin cDNA and long-term increase in serum levels of the protein (Stephens et al., 2018). Further studies are needed to characterize the immune response against CRISPR therapeutics delivered by adenoviral vectors.

Lentiviral vectors have the advantages of low immunogenicity and low prevalence of pre-existing immunity against them in the general population (Shirley et al., 2020; Dong and Kantor, 2021). Non-integrating lentiviruses have been developed to avoid viral integration into random sites in the host genome which carries the risk of disrupting the function of normal genes or causing cancer (Gurumoorthy et al., 2022). A non-integrating lentivirus delivering CRISPR/Cas9 has been successfully used for one-time correction of the sickle cell disease mutation in the β-globin gene (Uchida et al., 2021). Lentiviruses have been pseudotyped to alter their host range (Cronin et al., 2005; Humbert et al., 2012; Ebrahimabadi et al., 2019) and this can help increase their target cell specificity, reduce the dose, and minimize systemic immune responses. Transduction of antigen presenting cells by lentiviruses could be a limitation to the use of these vectors in the delivery of CRISPR therapeutics. This is known to promote a strong immune response against the transgene product (Shirley et al., 2020) and could thus increase the immune response to the CRISPR effector being delivered. One way to overcome this is to design the mRNA transcript to be recognized and degraded by miRNAs that are specifically expressed in hematopoietic cells but not in the target cell (Brown et al., 2006; Annoni et al., 2009). Non-viral delivery vectors are favored for their low immunogenicity, tissue targetability, versatile packaging capacity, and ease of manufacturing scale up (Wilbie et al., 2019). Lipid nanoparticles (LNPs) had been clinically used for the delivery of small interfering RNA (Thi et al., 2014) and mRNA Zika vaccine (Richner et al., 2017) prior to COVID-19 but gained particular attention following mass public vaccination with mRNA-LNP COVID-19 vaccines (Mulligan et al., 2020; Walsh et al., 2020). Several studies showed modest gene editing efficiencies and the need for multiple dosing upon delivery of Cas9 using non-viral vectors (Yin et al., 2016; Miller et al., 2017), potentially increasing the risk of immunogenicity of CRISPR components. It has been demonstrated, however, that the administration of a single dose of CRISPR/Cas9 delivered in LNPs resulted in robust and persistent gene editing for 12 months of transthyretin (Finn et al., 2018) and dystrophin (Kenjo et al., 2021) genes in mice. Dystrophin protein was shown to accumulate in mice following repeated administration of an LNP-CRISPR targeting skeletal muscle tissue while it failed to do so with AAV-CRISPR due to clearance by the host immune response (Kenjo et al., 2021). Repeated dosing could allow the use of lower doses which reduces the risk of eliciting an immune response.

### 5.5 Patient-specific considerations

As CRISPR moves to the clinic, important considerations related to the specific population that will receive the treatment need to be weighed in. CRISPR therapeutics may need to be personalized to each individual’s needs, immune status, pre-existing memory responses, treatment history, MHC polymorphism, and immunological risk. Not surprisingly, CRISPR trials in immunocompromised or immunosuppressed patients generally reported no or mild immune-related adverse reactions whether *ex vivo* (Xu et al., 2019; Lu et al., 2020; Stadtmauer et al., 2020; Uddin et al., 2020) or *in vivo* (Gillmore et al., 2021; Intellia Therapeutics, 2021; Intellia Therapeutics, 2022). For use in immunocompetent individuals, markers of potential immunotoxicity need to be identified based on individual MHC alleles and the specific CRISPR effector protein used. Two immunodominant and two subdominant MHC class I-restricted epitopes of SpCas9 have been identified for HLA-A*02:01 (Ferdosi et al., 2019). This was done using an enhanced prediction binding algorithm that takes into consideration both MHC binding affinity and T cell receptor contact amino acid hydrophobicity (Chowell et al., 2015), which showed superior prediction power to the commonly used IEDB analysis tool (Vita et al., 2015), as validated experimentally by T cell assays (Ferdosi et al., 2019). This approach was replicated by Shen et al. for SaCas9 B cell immunogenicity, resulting in reduced humoral immune recognition with a single amino acid substitution while retaining nuclease function (Shen et al., 2022). Targeted mutation to eliminate immunodominant epitopes markedly reduced immune recognition of SpCas9 while retaining its specificity as well as its nuclease and transcriptional modulation activities. Recently, 22 SaCas9-derived MHC class II-restricted epitopes that stimulate CD4^+^ cells have been identified using overlapping peptides that span the whole protein and mass spectrometry (Simhadri et al., 2021a). Enhanced epitope prediction models are currently only available for the most common MHC alleles and prediction algorithms that cover the diversity of MHC polymorphism are needed before universal use of deimmunized CRISPR effector proteins.

Another patient-specific factor that needs to be identified on an individual basis is the breadth and specificity of pre-existing immune responses to both CRISPR effectors and viral vectors, whether developed due to exposure to the source bacteria, cross-reactivity from a similar epitope, or treatment history. Pre-existing B cell and T cell responses to both SpCas9 and SaCas9 is widespread in the general population (Charlesworth et al., 2019; Ferdosi et al., 2019; Wagner et al., 2019; Shen et al., 2022) with a comparable frequency of pre-existing immune responses to CRISPR effectors from less common bacteria (Tang et al., 2022). Experience with the use of rAAV for gene therapy suggests that individual CRISPR-specific immune responses need to be considered. Individuals with pre-existing antibodies to AAV or who have previously received AAV-based gene therapy have been generally excluded from AAV clinical trials (Calcedo et al., 2009; Li et al., 2012). With a high proportion of the general population being seropositive to at least one AAV type and given the cross-reactivity between AAV types, both eligible individuals and repeated administration are greatly limited. The exponential growth of CRISPR pre-clinical studies and clinical trials in a relatively short amount of time (Adli, 2018; Katti et al., 2022) could mean that CRISPR therapeutics and even DIY CRISPR kits (Sneed, 2017) could become available to the public in the near future at least in some parts of the world. Specific immune responses to CRISPR effectors could become ubiquitous and therefore necessitate sufficient testing and personalized protein engineering based on individual immunoprofiling.

### 5.6 Intended CRISPR activity and duration

CRISPR-based gene editing has an advantage over traditional gene therapy approaches in that limited duration of action is sufficient for long-lived genetic modifications. This allows the design of CRISPR therapeutics that control CRISPR effector persistence and activity, which limits both off-target effects and immunogenicity. Cellular immune responses will thus be limited to the period during which CRISPR effector-derived immunogenic epitopes are displayed on the cell surface. Strategies for CRISPR control include the use of inducible promoters (Dow et al., 2015), anti-CRISPR proteins (Harrington et al., 2017; Davidson et al., 2020; Ibraheim et al., 2021), conditional CRISPR effector destabilization (Kleinjan et al., 2017; Senturk et al., 2017), and self-deleting AAV-CRISPR (Li et al., 2019). A self-limiting CRISPR-Cas9 system for LCA10 was developed with sgRNA recognition sites included on the Cas9 plasmid to allow plasmid destruction as soon as Cas9 is expressed. Even though Cas9 expression was transient, the targeted nuclease function was achieved and the levels of mutant mRNA were successfully reduced (Ruan et al., 2017).

While some therapeutic applications only require a single base substitution or homology-directed repair (HDR) with a short stretch of DNA, others necessitate the introduction of a functional copy of the defective gene. Targeted insertion of a transgene into the host genome can be mediated by HDR following double-strand break introduced by CRISPR at a specific genomic site (Hayashi et al., 2020; Nitzahn et al., 2021; Oh et al., 2022). This allows precise insertion at safe harbor loci to avoid disruption of normal gene functions. CRISPR/Cas9 has been used to treat ornithine transcarbamylase deficiency in mice through targeted insertion of the OTC gene, a broad approach that can be generalized regardless of the specific mutation that the patient carries (Wang et al., 2020). CRISPR/Cas9 has also been used for targeted knock-in of a CD19-specific chimeric antigen receptor resulting in CAR T cells with uniform CAR expression and improved potency compared with the conventional CAR T cell approach (Eyquem et al., 2017; Dimitri et al., 2022). Immune responses to the transgene product are a potential hurdle in the translation of CRISPR-based therapeutics intended for targeted insertion. This has been reported against α1-antitrypsin and dystrophin in AAV-based gene therapy clinical trials (Mendell et al., 2010; Calcedo et al., 2017). Several factors could contribute to the production of an immune response to the transgene product including the delivery vector, the specific mutation in the defective gene being corrected, and other host factors (Herzog, 2019). For example, viral vectors with higher transduction of APCs may result in a stronger immune response against the transgene product (Jooss et al., 1998).

Besides employing CRISPR effector protein nuclease activity for genome editing, there is a growing interest in the use of CRISPR-based synthetic transcription factors for *in vivo* control of gene expression and epigenome editing (Pandelakis et al., 2020). A modified CRISPR effector, whether nuclease-active or catalytically dead Cas9 (dCas9), is directed to the sequence of interest through a gRNA allowing epigenomic effector domains to bind to the target locus, resulting in targeted transcriptional modulation (Chavez et al., 2015; Dahlman et al., 2015; Kiani et al., 2015). The dynamics of the desired activation or repression depend on the type of cell and gene function, and thus dictate CRISPR dosage, intended persistence in the cells, and duration of activity, which potentially impact immunogenicity. Several strategies for spatiotemporal control of CRISPR-based transcriptional modulation have been developed including gene circuits (Kiani et al., 2014; Weinberg et al., 2017), optogenetic control (Putri and Chen, 2018), and drug-inducible systems (Gao et al., 2016). The immunogenicity of transcriptional modulatory CRISPR therapeutics will need to be elucidated *in vivo* for clinical translation to be feasible.

### 5.7 Treatment regimen

To avoid unfavorable immune responses, immunosuppressive drugs are often co-administered, a strategy employed by clinical trials using CRISPR (Gillmore et al., 2021), other gene therapy approaches (Corti et al., 2017; Mendell et al., 2017; Russell et al., 2017; Freitas et al., 2022), or cellular therapies (Ramzy et al., 2021; Shapiro et al., 2021). Corticosteroids are approved for use with two AAV-based gene therapy drugs in clinical use, Zolgensma (Mendell et al., 2017) and Luxturna (Russell et al., 2017). Methylprednisolone is used in combination with ciclosporin along with Glybera, another gene therapeutic in clinical use for lipoprotein lipase deficiency (Ferreira et al., 2014; Gaudet et al., 2016). Other immunosuppressant drugs such as rapamycin, ciclosporin, and tacrolimus are in various stages of pre-clinical and clinical development for use with AAV-based gene therapy (Gaudet et al., 2016; Corti et al., 2017; Biswas et al., 2020). With CRISPR therapeutics, cellular immune responses represent a major concern since CRISPR effectors are intended to be intracellularly expressed. Thus, immunosuppressants that inhibit T cell responses may represent attractive approaches for use with CRISPR gene editing, at least transiently. Different combinations of immunosuppressants can also be considered (Xiang et al., 2022). Recently, biodegradable nanoparticles encapsulating rapamycin (ImmTOR) were shown to mitigate the immunogenicity of biologics both in preclinical (Kishimoto, 2020; Ilyinskii et al., 2021) and clinical (Sands et al., 2022) trials, while free rapamycin was only partially effective (Shi et al., 2021). However, while immune suppression may allow re-dosing in naïve individuals, it is unlikely to circumvent pre-existing immune responses in individuals who have been previously exposed.

Alternatively, adoptive transfer of CRISPR effector-specific T_reg_ cells following *ex vivo* expansion can be used to induce tolerance to CRISPR therapeutics in humans. Adoptive T_reg_ cell therapy is in clinical trials for the prevention of graft versus host disease in transplantation and the treatment of type 1 diabetes (Terry and Oo, 2020; Dong et al., 2021) and has been previously shown to mitigate cellular immunity in gene therapy in preclinical studies (Sarkar et al., 2014). Wagner et al. (2019) showed that SpCas9-specific T_reg_ cells suppressed effector T cells that recognize SpCas9 with suppression of cytokine production in cocultures. The authors note that T_reg_ and T_eff_ cells are possibly specific to different regions of the Cas9 protein, which can be harnessed for either *ex vivo* or *in vivo* generation of CRISPR effector-specific T_reg_ but not T_eff_ cells (Wagner et al., 2019; Wagner et al., 2021b).

## 6 Monitoring the immune response to CRISPR therapeutics

As with other gene therapy approaches, monitoring both pre-existing and therapy-induced immune responses may eventually be a routine component of clinical practice involving CRISPR therapeutics. There has been limited regulatory guidance on appropriate immunogenicity assessment protocols primarily due to limited availability of relevant data. There is a need to incorporate thorough investigation of pre-existing and emergent immunity to CRISPR therapeutics in the design of clinical trials to better understand its significance and potential clinical consequences. Regulatory guidance in the last few years has focused on *ex vivo* approaches in which exposure of the immune system to CRISPR components is limited (Piccoli et al., 2019). With the rise in the number of clinical trials using an *in vivo* approach, the evaluation of biodistribution of CRISPR components and the extent of immune recognition is increasingly needed for both *ex vivo* and *in vivo* treatments (Loo et al., 2022).

Currently, clinical trials that use AAV vectors for transgene delivery commonly screen participants for pre-existing immunity to exclude individuals who are positive or have high antibody titers (Verdera et al., 2020). Participants with history of antibodies to the transgene product such as individuals with FVIII inhibitors in hemophilia, are also excluded (Long et al., 2021). Similarly, investigation of the immune response to CAR T cell therapy is recommended in phase I clinical trials (Wagner et al., 2021a; Loo et al., 2022), particularly after lethal anaphylaxis due to human anti-mouse antibodies (HAMA) was reported (Maus et al., 2013). For CRISPR therapeutics, as discussed above, the design of immunogenicity risk assessment protocols will depend on the clinical setting and intended application.

**FIGURE 2:**
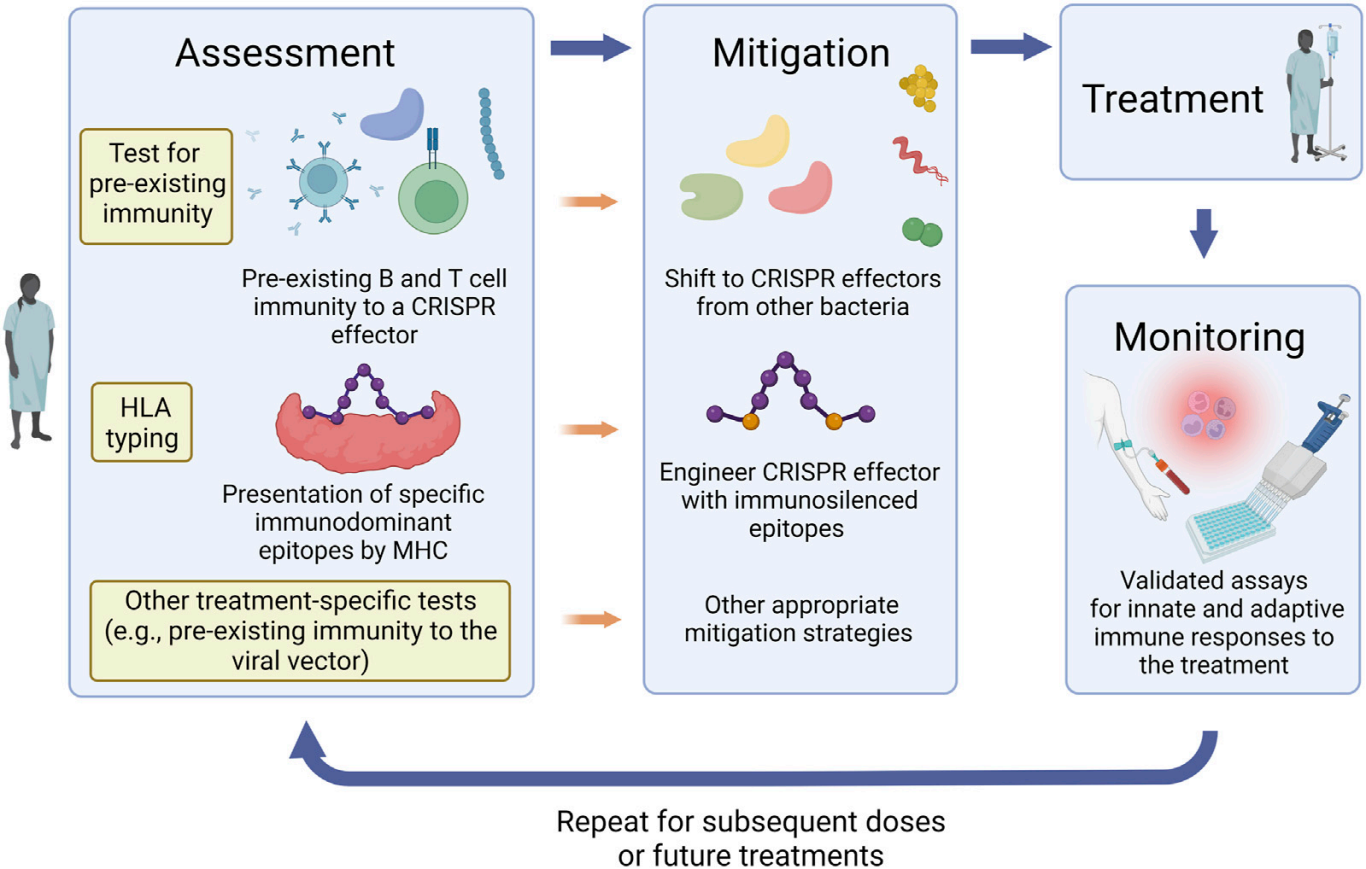
Assessment, mitigation, and monitoring of immune responses to CRISPR therapeutics. Strategies for mitigation of immune responses can be personalized to each patient’s needs, history, immune status, and treatment plan. Pre-treatment assessment protocols can include screening for immune responses to different CRISPR effector proteins, HLA typing, and other tests that are relevant to the treatment approach such as pre-existing immune responses to the viral vector if one is used for delivery. According to the assessment results, appropriate mitigation strategies can then be implemented. These can include selection of a CRISPR effector ortholog which the patient has no pre-existing immunity against and engineering the selected protein to silence the immunodominant epitope(s) based on the individual’s MHC haplotype. Following administration of the CRISPR therapeutic, the patient should be monitored for the development of innate or adaptive immune responses and appropriate intervention strategies can be implemented accordingly. The assessment process can be repeated for each subsequent dose or for future administration of a different CRISPR therapeutic.

The aforementioned considerations can help guide the design of guidelines for appropriate assessment, monitoring, and mitigation of immune responses to CRISPR therapeutics. For instance, evaluation of pre-existing and treatment-induced specific antibodies in vitreous fluid can be implemented with treatments involving local subretinal delivery of CRISPR components. Testing for pre-existing and treatment-emergent immune responses to the viral vector will be required whenever a viral vector is used for delivery. When a patient is considered for treatment, assessment and mitigation strategies can be personalized to their individual needs (Figure 2). Individuals receiving the treatment can be initially screened for immune responses to different CRISPR effector proteins for selection of an ortholog with no pre-existing immunity against. The selected protein can then be engineered to silence the immunodominant epitope(s) based on the individual’s MHC haplotype. This personalized approach is not currently approved by the FDA. An alternative solution is to obtain individual FDA approvals for MHC haplotype-specific CRISPR effector variants designed for use in patients with specific HLA alleles. Following administration, the patient will be monitored for the development of innate or adaptive immune responses and appropriate intervention strategies can be implemented accordingly. The assessment process can be repeated for each subsequent dose or for future administration of a different CRISPR therapeutic.

There is a need for validated assays to assess the immunogenicity of CRISPR therapeutics in various stages of preclinical and clinical development. The development of Clinical Laboratory Improvement Amendments (CLIA)-based assays for use in clinical trials necessitates the development of clinical laboratory grade reagents and assay validation and harmonization. It has been recently reported that commercial Cas9 protein from seven manufacturers contains varying levels of endotoxin (Simhadri et al., 2021b). This can lead to false positive results in T cell assays that use the whole protein, especially that commercial Cas9 was not designed for the purpose of assessing immunogenicity. To circumvent this limitation in a research setting, T cell immune responses to Cas9 can be measured using synthetic predicted peptides or antigen presenting cells expressing the CRISPR effector under study (Ferdosi et al., 2019). For clinical settings, the development and validation of standardized clinical assays and reagents will be of paramount importance for clinical translation of CRISPR therapeutics.

## 7 Conclusion

Rapid advances in both preclinical and clinical development of CRISPR-based therapeutics suggest that the approval of CRISPR drugs for clinical use might be anticipated in the near future. Several challenges that could potentially hold the field back remain to be addressed, including the immunogenicity of CRISPR components. Optimization of CRISPR therapeutics to have no or low immunogenicity with the intended therapeutic application needs to be implemented early in the development pipeline for proper evaluation of safety and efficacy. One or more mitigation approaches may be needed depending on the disease being treated, patient population, treatment strategy, and other considerations identified here. Further research is needed to address knowledge gaps and ensure the delivery of safe and effective CRISPR-based therapeutics.
